# Development of a real-world database for asthma and COPD: The SingHealth-Duke-NUS-GSK COPD and Asthma Real-World Evidence (SDG-CARE) collaboration

**DOI:** 10.1186/s12911-022-02071-6

**Published:** 2023-01-09

**Authors:** Sean Shao Wei Lam, Andrew Hao Sen Fang, Mariko Siyue Koh, Sumitra Shantakumar, See-Hwee Yeo, David Bruce Matchar, Marcus Eng Hock Ong, Ken Mei Ting Poon, Liming Huang, Sudha Harikrishan, Dominique Milea, Des Burke, Dave Webb, Narayanan Ragavendran, Ngiap Chuan Tan, Chian Min Loo

**Affiliations:** 1 Health Services and Systems Research, Duke-NUS Medical School, Singapore, Singapore; 2 Health Services Research Centre, Singapore Health Services, 20 College Road, The Academia – Discovery Tower Level 6, Singapore, 169856 Singapore; 3 Health Services Research Institute, SingHealth Duke NUS Academic Medical Centre, Singapore, Singapore; 4 Lee Kong Chian School of Business, Singapore Management University, Singapore, Singapore; 5 SingHealth Polyclinics, SingHealth, Singapore, Singapore; 6 Department of Respiratory and Critical Care Medicine, Singapore General Hospital, Singapore, Singapore; 7 Duke-NUS Medical School, Singapore, Singapore; 8 GlaxoSmithKline, Singapore, Singapore; 9 Department of Internal Medicine (General Internal Medicine), Duke University Medical School, Durham, NC USA; 10 Department of Internal Medicine, Singapore General Hospital, Singapore, Singapore; 11 Department of Emergency Medicine, Singapore General Hospital, Singapore, Singapore; 12 Integrated Health Information Systems, Singapore, Singapore

**Keywords:** Real world data, Real world evidence, Database, Asthma, Chronic obstructive pulmonary disease, COPD

## Abstract

**Purpose:**

The SingHealth-Duke-GlaxoSmithKline COPD and Asthma Real-world Evidence (SDG-CARE) collaboration was formed to accelerate the use of Singaporean real-world evidence in research and clinical care. A centerpiece of the collaboration was to develop a near real-time database from clinical and operational data sources to inform healthcare decision making and research studies on asthma and chronic obstructive pulmonary disease (COPD).

**Methods:**

Our multidisciplinary team, including clinicians, epidemiologists, data scientists, medical informaticians and IT engineers, adopted the hybrid waterfall-agile project management methodology to develop the SingHealth COPD and Asthma Data Mart (SCDM). The SCDM was developed within the organizational data warehouse. It pulls and maps data from various information systems using extract, transform and load (ETL) pipelines. Robust user testing and data verification was also performed to ensure that the business requirements were met and that the ETL pipelines were valid.

**Results:**

The SCDM includes 199 data elements relevant to asthma and COPD. Data verification was performed and found the SCDM to be reliable. As of December 31, 2019, the SCDM contained 36,407 unique patients with asthma and COPD across the spectrum from primary to tertiary care in our healthcare system. The database updates weekly to add new data of existing patients and to include new patients who fulfil the inclusion criteria.

**Conclusions:**

The SCDM was systematically developed and tested to support the use RWD for clinical and health services research in asthma and COPD. This can serve as a platform to provide research and operational insights to improve the care delivered to our patients.

**Supplementary Information:**

The online version contains supplementary material available at 10.1186/s12911-022-02071-6.

## Introduction

Real-world data (RWD) in healthcare refers to data that are routinely collected as part of the care delivery process, rather than through clinical trial settings. RWD can be used to generate real-world evidence (RWE) [1]. The potential uses of RWE are broad, ranging from clinical guidelines development to enabling precision medicine in clinical practice [2–4]. With the adoption of electronic health records (EHR) and recent legislations such as the 21st Century Cures Act [5], there has been an increasing interest in using real-world evidence (RWE) to satisfy the needs of the evolving healthcare industry [5, 6]. Various initiatives have been organized around the use of RWE, such as the Duke-Margolis Centre for Health Policy RWE Collaborative, to advance policy development related to regulatory acceptability of RWE [7]. RWE has successfully been used by the US Food and Drug Administration in its approval of a cancer therapy drug label expansion in April 2019 [8].

Obtaining RWD from information systems can be done manually or automatically. Manual extraction entails visual inspection of patient records and manual transcription. Such methods are laborious and vulnerable to transcription errors [9]. Given these issues, researchers have increasingly relied on the automated methods for data collection [10–12]. This allows for efficient, near real-time research on clinical practice, while minimizing the risk of data entry errors.

There have been a number of well-reported large-scale RWD for various clinical care domains, for example, the Clinical Practice Research Datalink (CPRD) which is a primary care database of anonymized medical records [13], European Severe Heterogeneous Asthma Registry, Patient-centred (SHARP) Clinical Research Collaboration [14], UK Severe Asthma Registry (UKSAR) [15], US Advancing the Patient EXperience (APEX) in Chronic Obstructive Pulmonary Disease (COPD) [16] registry amongst others.

In Singapore, a public–private sector collaboration—the SingHealth-Duke-GlaxoSmithKline COPD and Asthma Real-World Evidence (SDG-CARE) collaboration—was formed in 2017 to accelerate the use of RWD. With the above in mind, the collaboration aimed to develop a near real-time integrated RWD database—the SingHealth COPD and Asthma Data Mart (SCDM). The RWD is updated every 24 h, thereby providing a near real-time basis for effectively querying updated clinical and operational data. This is the first large-scale registry in Singapore to fully realize the potential of RWD to improve the care of patients with COPD and asthma. The SCDM is intended to be sufficiently robust to support the conduct of most clinical and health services research trials surrounding asthma and COPD, while ensuring minimal intrusion via the electronic medical record (EMR) systems. This study describes the development of the SCDM and provides an overview of its contents.

## Methods

### Setting, systems and stakeholders

SingHealth is the largest of the three public health systems in Singapore, and consists of public hospitals, community hospitals, national specialty centers and a network of eight primary care clinics (polyclinics). Singhealth provides medical care to over 2 million patients in this city-state of 5.8 million population and attracts patients from all over the country [17, 18]. For this collaboration, two SingHealth clinical sites, Singapore General Hospital (SGH) and SingHealth Polyclinics (SHP) were involved. SGH is a tertiary multispecialty academic hospital with 1,785 beds and provides specialist care to over 1 million patients a year, and SHP is a primary care network of 8 clinics that caters to about 2 million patient attendances a year [17].

**Fig. 1:**
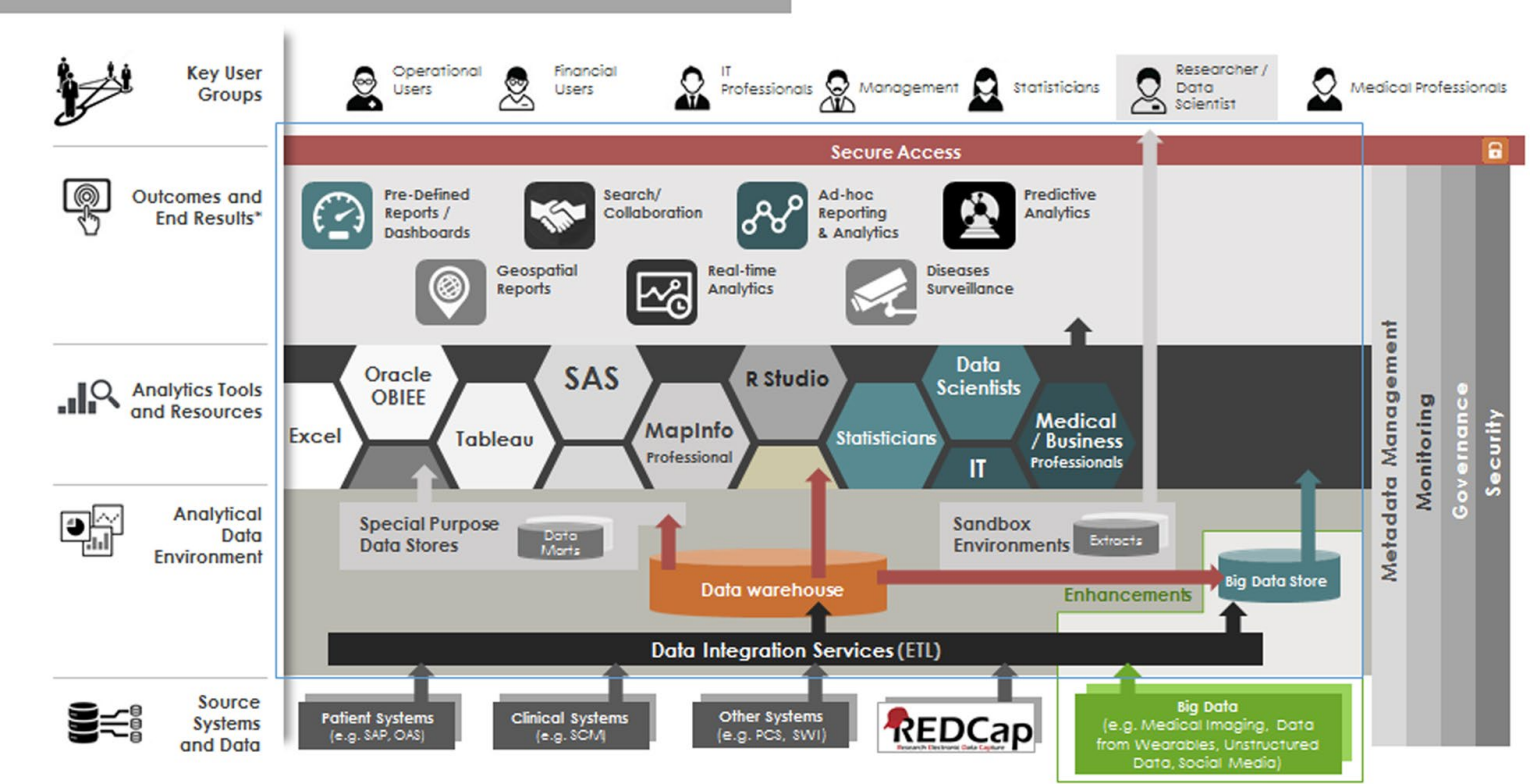
Overview of analytics support infrastructure in SingHealth. *Note* Electronic Health Intelligence System (eHints) [19] is the enterprise data warehouse for SingHealth. It integrates data from various IT systems and feeds them into analytics tools for research and clinical care

Over the years, SingHealth has established a comprehensive integrated enterprise information technology (IT) system that supports a broad range of functions ranging from administrative to clinical and operational functions. A core component of the SingHealth IT and data infrastructure is her enterprise data warehouse (EDW)—SingHealth Electronic Health Intelligence System (eHints) [19]. Data from various clinical, operations and research sources are ingested into eHints automatically through an Informatica-based [20] Extract-Transform-Load (ETL) layer. Data in eHints can be organized into data marts to orientate to specific domains (e.g. finance) and subject areas. Once the data is consolidated in the EDW, it can then be consumed through the Oracle Business Intelligence Enterprise Edition (OBIEE) analytics platform [21, 22] to support advanced, near real-time user reporting, dash-boarding and other important enterprise business intelligence functions (Fig. 1).

Prior to the development of the SCDM, mainly the administrative and operational systems were integrated with eHints. For the development of the SCDM, various standalone clinical systems had to be newly integrated. One of the key clinical systems used in SingHealth is Sunrise Clinical Manager™ (SCM) [23], a commercial electronic medical records (EMR) system by Allscripts (Allscripts Healthcare LLC).

The administration and maintenance of most IT systems for the public healthcare system is under the purview of Integrated Health Information System (IHiS) [24]. This includes the OBIEE platform. IHiS is a distinct IT organization who engages in a client-vendor relationship with SingHealth. Given the engagement framework, there is a need to predict the manpower capacity that is required, and clear metrics for monitoring project progress (via planned milestones) [25]. However, the dynamic and uncertain requirements inherent in the design of a registry which leverages on clinical and operational data requires flexibility in requirement changes. There is thus a need for short feedback cycles with close stakeholder engagement. The organizational setup and project requirements dictate the need for a hybrid project management methodology which leverages on well-planned waterfall methodologies coupled with sub-modules which are executed in an agile approach with close stakeholder engagement across each of the sub-modules [26, 27]. The submodules ensured that correct data sources are ingested into the data warehouse and properly transformed and standardized prior to each milestone.

The SCDM was designed and developed with the involvement from clinicians, medical informaticians, IT engineers and project managers from SingHealth, IHiS and GlaxoSmithKline (GSK). It was built within the SingHealth eHints platform [19] and governed in compliance to all existing cybersecurity and privacy laws for the healthcare sector in Singapore [28]. SCDM is under the ownership of SingHealth, and the custodianship of the SDG-CARE Steering Committee.

In developing the SCDM, the team complied with all applicable laws regarding patient privacy. Ethics board approval was obtained as part of the SDG-CARE collaboration, prior to developing the SCDM (SingHealth Centralized Institutional Review Board Ref No. 2017/2950).

A study protocol was also produced to clearly define the objectives and deliverables of the SDG-CARE collaboration. The SCDM was developed in accordance with this study protocol.

### Development of the SCDM ETL algorithm

To ensure a comprehensive and systematic approach, the team adopted a hybrid waterfall-agile methodology in developing the SCDM. Waterfall methodology is a linear project management approach where stakeholder and customer requirements are gathered at the beginning of the project, and a sequential project plan is then created to accommodate those requirements. The agile methodology was used for rapid reviews with frequent stakeholders’ engagement sessions to derive the unified data model within the design and development phase. The following details the broad phases.

1. Requirement gathering

This was a critical step in the waterfall aspect of the hybrid methodology where requirements were gathered, allowing other phase to be planned. To do this, the task of data profiling was undertaken. Data profiling involved first listing down the source IT systems that captured asthma and COPD relevant data (e.g. EMR, radiology information system, outpatient administration system) and then reviewing the list of variables captured in each of these systems. Face-to-face requirement gathering sessions with the various stakeholders (i.e. clinicians, researchers, medical informaticians and IT engineers) were conducted to frame the high level scope of work followed by deep diving into detailed data requirements. Clinicians and medical informaticians reviewed screen shots of each end user EMR screen to select required front-end data fields. Based on these requirements, IT engineers then identified the matching back-end data sources and assessed the feasibility of extracting the data. At the end of this phase, a detailed user requirements document (URD) was compiled to formalise the business requirements for IT implementation. The URD specified clearly the initial data elements to be captured in the SCDM.

2. Design and development

The purpose of the design phase was to define the data mart schema and to create an ETL specification document. The overall SCDM ETL mechanism was designed as a two-step process to mirror typical research study protocols. The first ETL step involved identifying a cohort of patients who have asthma and COPD based on a set of pre-defined inclusion criteria, followed by importing their pre-selected data elements. To identify patients for inclusion in the SCDM, the team used a Place-Diagnosis-Time framework to define a multidimensional inclusion criterion. The “Place” component refers to the visit location (i.e. SGH or SHP). The “Diagnosis” component refers to the diagnosis for the visit (e.g. asthma or COPD), and the “Time” component refers to the date of the visit (i.e. whether it falls within a specified time window). In the interest of keeping the SCDM robust, no exclusion criteria were used.

The selected data elements to import were captured in the URD. As there were common data elements captured in SGH and SHP that were labelled and stored differently in the back-end databases, the agile method was also used across several scrum cycles to resolve the data differences with the stakeholders. These were mapped into unified data elements in the SCDM.

The developers translated the ETL document to actual Informatica ETL codes. The OBIEE subject areas were also developed. Test cases and scripts were then created to facilitate system integration testing by the IT engineers. Upon the completion of the SCDM, the SCDM ETL mechanism design was compiled into an ETL document to provide developers with a lineage of each data element. The design of the user interface based on the OBIEE platform was also documented.

3. User acceptance test (UAT)

In this phase, the business stakeholders (i.e. clinicians, researchers and medical informaticians) reviewed the system to ensure that it met the requirements laid out at the beginning of the project. This was done by releasing a completed product for testing and verification.

A UAT briefing was conducted by the system developers to guide users on how to access the SCDM via OBIEE. A UAT test plan and test cases were also mutually agreed between SingHealth and IHiS to ensure all stakeholders were aligned on the project exit criteria. UAT was conducted in two phases to adhere with organizational policy which directed that production data should not be used for testing purposes in test environment. Phase 1 was a functional test where users focused on testing that front-end interfaces were in accordance with requirements in test environments. Phase 2 focused on data verification where users compared data from SCDM and source systems in the production environments.

For Phase 2 of the UAT, three team members from SingHealth verified the data extracted from SCDM with data in the EMR systems. There were two testing sub-components, which mirrored the two steps in the ETL mechanism. In the first step, the testers would check that the cohort extracted from SCDM matched the cohort extracted from the EMR database using identical extraction criteria. In the next step, all data elements of a 100 patients sample from the SCDM were extracted. These were then manually checked against their data in the HER system. Finally, aggregated data from SCDM was computed and compared with published data from the same population.

Once the UAT was complete, the testers signed off on a UAT document and a deployment checklist was prepared for system go-live.

4. Implementation and post-implementation support

Upon user acceptance, the SCDM was deployed in a production environment with the necessary rectification identified during UAT. Subsequently, IHiS provided technical support to users. A data dictionary was produced to facilitate understanding of the various data elements in the SCDM. A user manual was also produced to explain to users the SCDM’s applicability and to provide step-by-step instructions for data extraction.

## Results

### Overview

**Fig. 2:**
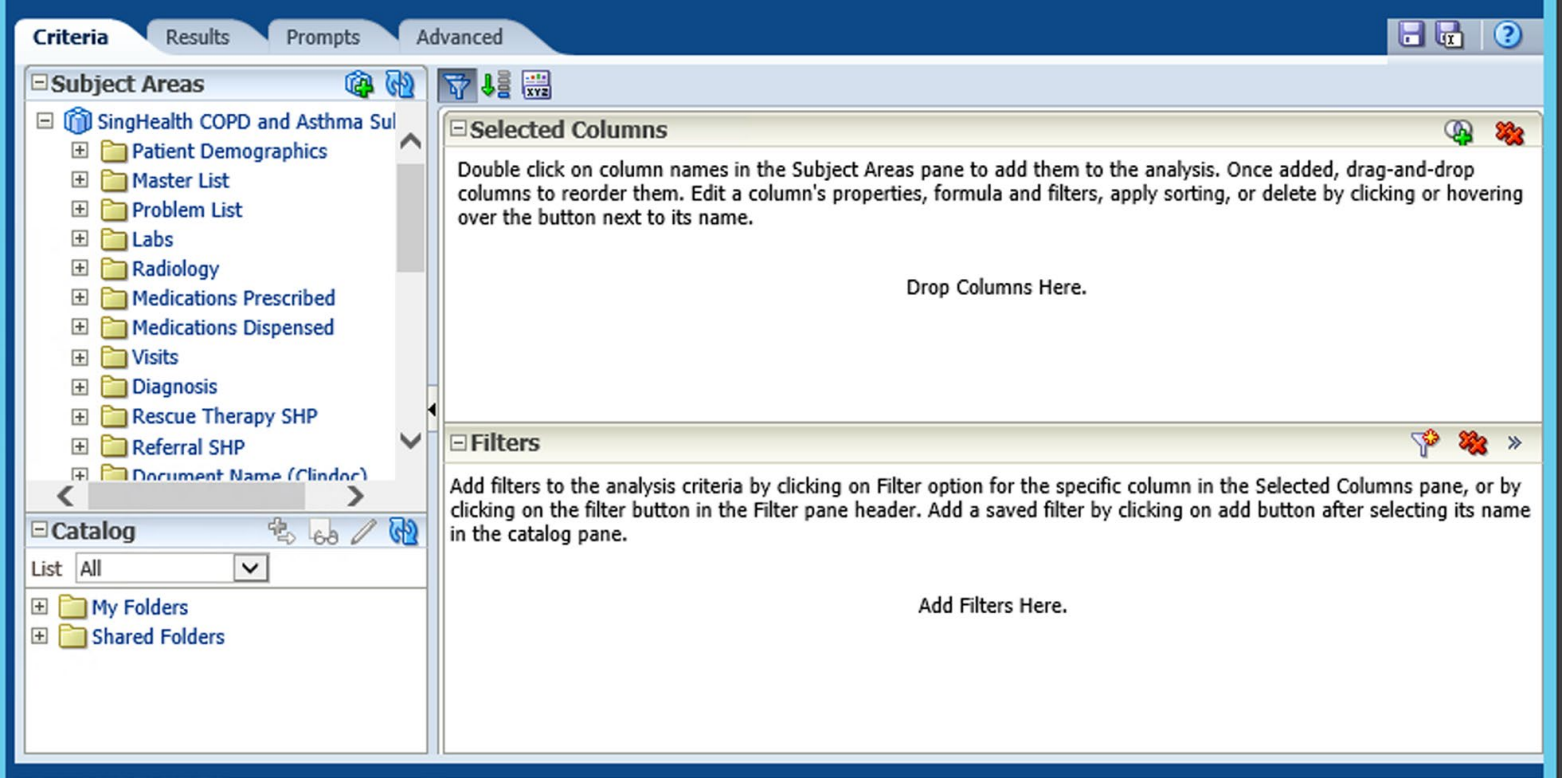
Screen capture of SingHealth COPD and Asthma Data Mart (SCDM) user interface in eHints. *Note* The Oracle Business Intelligence Enterprise Edition (OBIEE) analytics platform is the front-end of Electronic Health Intelligence System (eHints), allows users to drag-and-drop columns for data extraction, instead of having to write SQL codes. It is meant to support self-service data extraction

The SCDM is a unified data repository within eHints which integrates data from various source systems. Data in SCDM is updated in batches on a weekly basis, where data of existing patients is updated and new patients are added. It is accessible via OBIEE which has a friendly user interface to supporting drag-and-drop to enable reporting and analysis for business intelligence (Fig. 2).

SCDM’s cohort definition is based on patients having at least one of the pre-defined diagnosis codes recorded in the SCM clinical document when they visit the SGH Department of Respiratory and Critical Care Medicine (RCCM) or SHP on or after January 1, 2015 up to current date.

The pre-defined diagnosis codes (with SNOMED-CT Description ID) are listed below:

- Allergic bronchopulmonary aspergillosis (63349014)
- Aspirin exacerbated respiratory disease (3038385014)
- Asthma (301485011)
- Asthma-COPD overlap syndrome (ACOS) (3046475015)
- Bronchiectasis (21163015)
- Chronic bronchitis (105519017)
- Chronic obstructive pulmonary disease (475431013)
- Churg-Strauss syndrome (136476013)
- COPD—Chronic obstructive pulmonary disease (475427019)
- Severe asthma (1208972017)

There are a total of 199 data elements organized into 28 folders within a single subject area. Table 1 lists the 28 folders, while the list of 199 data elements can be found in the Additional file 1: Table 1. In some cases where the same elements were available from both SGH and SHP, these elements were mapped and reconciled.

**Table 1:** SingHealth COPD and Asthma Data Mart (SCDM) Folders

No	Folder
1	Master List^1^
2	Patient Demographics
3	Problem List^2^
4	Laboratory Results
5	Radiology Reports
6	Medications Prescribed
7	Medications Dispensed
8	Visit Details^3^
9	Diagnosis^4^
10	Rescue Therapy^5^
11	Referrals^6^
12	Clinical Document Metadata
13	Patient History^7^
14	Objective Diagnosis
15	Asthma Control Test
16	COPD Assessment Test
17	Modified Medical Research Council Dyspnea Scale
18	GOLD Score
19	Physical Measurements
20	Physical Examination^7^
21	Peak Expiratory Flow Rate
22	Spirometry Results
23	Smoking Status
24	Vaccination Details^8^
25	Written Asthma Action Plan
26	Management Plan^7^
27	Asthma Counselling
28	Pulmonary Rehabilitation

*COPD* Chronic obstructive pulmonary disease, *GOLD* Global Initiative for Obstructive Lung Disease

^1^The data extraction is done in a two-step process. Firstly, the Master List is used to extract the ID for patients of interest. Then with this list of IDs, the other subject areas are used to extract the rest of the data elements of interest

^2^Problem list conditions were based on SNOMED Clinical Terms (SNOMED-CT) coding

^3^Visits details includes details such as visit date, visit location, and visit provider

^4^Diagnosis conditions were based on 10th revision of the International Classification of Diseases (ICD-10) coding

^5^Rescue therapy is a protocol-based bronchodilator intervention administered at the polyclinic for patients assessed to have asthma exacerbation

^6^Referrals made from SingHealth Polyclinics to tertiary hospitals, including Singapore General Hospital

^7^Patient history, physical examination findings and management plan were captured as free-text data

^8^For influenza and pneumococcal vaccinations only

### Data verification

For Phase 2 UAT, a retrospective data extraction was performed from both the EMR and SCDM using the following extraction criteria: (1) At least one visit to SGH RCCM specialist clinics and/or SHP, and (2) for asthma or COPD, and (3) between January 1, 2019 and December 31, 2019.

19,434 patients were found in both the EMR and SCDM datasets in that period of time. 4 patients were in the EMR dataset, but absent in the SCDM dataset, while there were no patients in the SCDM dataset that were absent in the EMR dataset. The discrepancies were shared with the IT team. Thorough investigation was conducted and it was found that the discrepancies were due to residual dummy cases used for system testing. In other words, the precision and recall of the ETL mechanism in identifying patients were both 100%.

For data element verification of the 100 sample patients, data extracted from the SCDM for each patient was prepared into a structured form and then manually compared with data displayed on the EMR system. Agreement rate of the SCDM data import mechanism was computed using EMR data as the reference. The agreement rate of the data elements checked for the 100 randomly sampled patients was 100% for all 27 categories except for Problem List and Prescribed Medications (Table 2). These errors were deemed non-critical. They included the importing of a cancelled medication, not including the free-text remarks available for some medications and not including comorbidities data entered before year 2015.

**Table 2:** Summary of data import mechanism for the sampled SingHealth COPD and Asthma Data Mart (SCDM) patient subset

Subject area	Observed (count)	Agreement rate^1^ (%)
Patient Demographics	100	100
Problem List	99	98.99
Laboratory Results	83	100
Radiology Reports	68	100
Medications Prescribed^2^	100	90
Visit Details	100	100
Rescue Therapy	30	100
Referrals	71	100
Clinical Document Metadata	100	100
Patient History	99	100
Objective Diagnosis	2	100
Asthma Control Test	69	100
COPD Assessment Test	1	100
Modified Medical Research Council Dyspnea Scale	1	100
Global Initiative for Obstructive Lung Disease Score	1	100
Physical Measurements	65	100
Physical Examination	80	100
Peak Expiratory Flow Rate	55	100
Spirometry Results	6	100
Smoking Status	68	100
Vaccination Details	37	100
Written Asthma Action Plan	74	100
Management Plan	92	100
Asthma Counselling	4	100
Pulmonary Rehabilitation^3^	0	NA

^1^Agreement rate is the number of matching SCDM and EMR data fields with observed data divided by the number of EMR data fields with observed data

^2^The agreement rate for prescribed medications was lower than other datasets because of the import of a cancelled medication, and not including the free-text remarks for some other medications

^3^For pulmonary rehabilitation, there was no observed data in the 100 sampled patients. A deliberate exercise to search for a case with observe data was conducted and was found to have matching data in the data mart

Finally, the team cross-checked aggregated data from SCDM with published data by Zheng et al. [29] and Tay et al. [30] on the same polyclinic and tertiary care populations. Comparing the numbers, as shown in Tables 3 and 4, found them to be largely similar.

**Table 3:** Comparison of data from SingHealth COPD and Asthma Data Mart (SCDM) SGH asthma cohort and asthma cohort from Tay et al. [30]

	Comparison Cohort (Tay et al. [30]) n = 420	SCDM SGH Asthma cohort (2015 to 2019) n = 5,563
Age, mean (SD) years	52 (18)	52.1 (21.6)
Gender = Male, %	47.1	51.0
Race, %		
Chinese	56.2	58.9
Malay	24.8	14.9
Indian	13.3	16.7
Others	5.7	9.4
Ex or current smoker*, %	24.3	15.3
Pre-bronchodilator FEV1 percentage^1^, mean (SD)	76 (23)	77.0 (3.0)

^1^Excluding those with missing values in computation of proportions and mean. Most of the analysis matched the existing studies except for the higher proportion of current or ex-smokers (24.3% vs 15.3%) and the higher proportion of Malays in the test cohort from Tay et al. [30] (this could be because the test cohort used by Tay et al. [30] included an external set of patients)

**Table 4:** Comparison of data from SingHealth COPD and Asthma Data Mart (SCDM) SHP asthma cohort and asthma cohort from Zheng et al. [29]

	Comparison Cohort (Zheng et al. [29]) n = 14,755	SCDM SHP Asthma cohort (2015 to 2019) n = 29,574
Average number of attendances for asthma per year	35,731	33,805
Gender = male % by attendance	39.7	42.7
Race, % by attendance		
Chinese	53.2	53.2
Malay	26.2	25.5
Indian	14.0	14.0
Others	6.6	7.3
Proportion of attendances with good asthma control (ACT ≥ 20), %	80.9	83.1
Proportion of attendances with rescue therapy^1^, %	11.7	8.8

^1^Most of the findings match except for the proportion of attendances with rescue therapy. This may be attributed to the different attendance years being evaluated in each of the cohorts

### Data contents and ETL design

The ETL extracted data from the Sunrise Clinical Manager™ system [23] across the following data sources (actual data source names have been amended for clarity):

- Respiratory Medicine Consult Notes
- Respiratory Medicine Follow-up Consult Notes
- Respiratory Medicine Assessment Notes
- Respiratory Medicine Asthma Consult Notes
- Respiratory Medicine COPD Consult Notes
- Family Medicine Clinical Notes

**Fig. 3:**
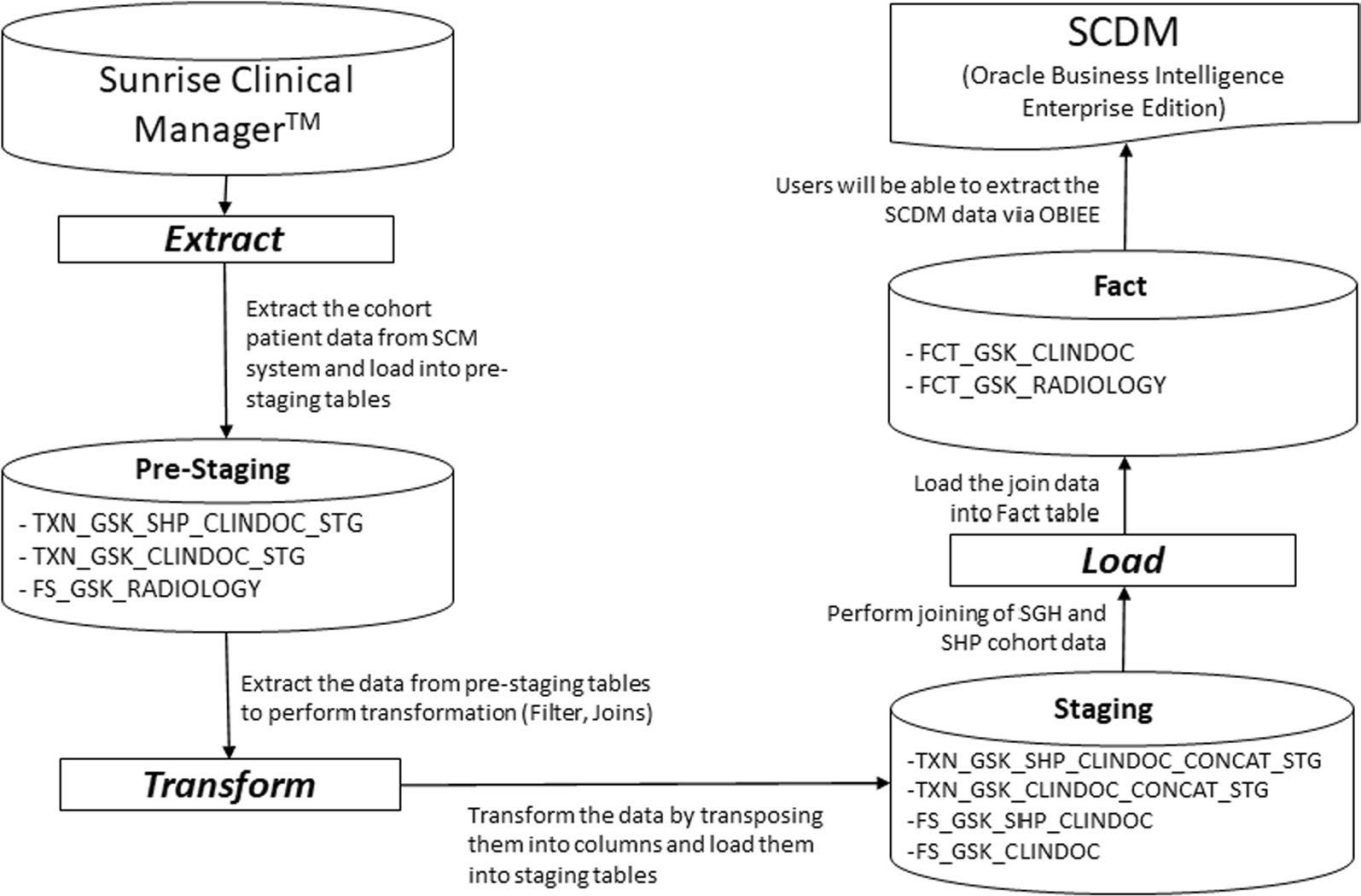
ETL Process for data sources from both SGH and SHP (target data tables are listed in the intermediate ETL steps)

The extracted data is then loaded into pre-staging, staging and fact tables through the ETL process shown in Fig. 3. Once the patients are recruited into the cohort based on the inclusion and exclusion criteria, retrospective data will be streamed into the ETL pipeline. For new patients who are recruited into the cohort, retrospective data will be brought into the SCDM every 24 h. For existing patients, their data will be incrementally loaded every 24 h.

**Fig. 4:**
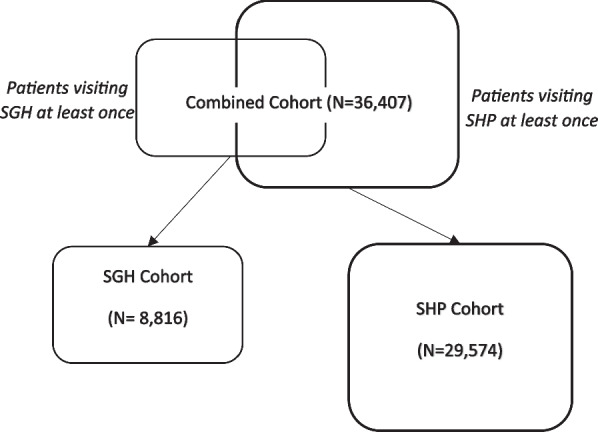
Cohorts used in the preliminary analysis—Combined, SGH and SHP. *Note* There are some patients who would fall under both circles when the cohorts are combined, hence the lower number in the combined cohort than compared to adding the individual SGH and SHP cohorts

A high-level cohort analysis was done to provide a summary of the data within SCDM for patients recruited into the cohort. In total, there were 36,407 patients in the SCDM as of December 31, 2019. Figure 4 illustrates how the various cohorts were composed for the analysis, while Table 5 provides a summary of the data extracted for these patients.

**Table 5:** High-level summary of data in the SingHealth COPD and Asthma Data Mart (SCDM) as of 31-Dec-2019

	SGH cohort n = 8,816	SHP cohort^1^ n = 29,574	Combined cohort n = 36,407
Age^2^, mean (SD) years	59.1 (20.9)	50.3 (23.0)	51.8 (22.9)
Gender = male, %	54.5	50.9	51.6
Race			
Chinese	5,950 (67.5)	15,062 (50.9)	19,749 (54.2)
Malay	1,047 (11.9)	8,499 (28.8)	9,250 (25.4)
Indian	1,127 (12.8)	3,729 (12.6)	4,550 (12.5)
Others	692 (7.8)	2,284 (7.7)	2,858 (7.9)
Smoking habit^3^, n (%)			
Non-smoker	3,073 (34.9)	18,650 (63.0)	20,208 (55.5)
Smoker	435 (4.9)	1,171 (4.0)	1,469 (4.0)
Ex-smoker	355 (4.0)	169 (0.6)	440 (1.2)
Not available	4,953 (56.2)	9,584 (32.4)	14,290 (39. 3)
Condition, n (%)			
Asthma	5,257 (59.6)	25,896 (87.6)	29,730 (81.7)
COPD	1,411 (16.0)	3,021 (10.2)	4,057 (11.1)
Both	306 (3.5)	657 (2.2)	778 (2.2)
Neither^4^	1,842 (20.9)	0 (0.0)	1,842 (5.0)
Year entered SCDM, n (%)			
2015	1,969 (22.3)	13,885 (47.0)	14,837 (40.8)
2016	2,020 (22.9)	4,915 (16.6)	6,530 (17.9)
2017	1,635 (18.5)	3,790 (12.8)	5,159 (14.2)
2018	1,732 (19.7)	3,763 (12.7)	5,271 (14.5)
2019	1,460 (16.6)	3,221 (10.9)	4,610 (12.7)
Total number of visits^5^	38,773	198,047	236,820

^1^The SHP cohort includes the pediatric population

^2^Refers to age at entry into the SCDM

^3^Based only on data captured in structured data input fields, excluding smoking data captured in free-text fields

^4^Of the ten pre-defined diagnosis codes used for inclusion into SCDM, some were technically not asthma or COPD diagnoses (e.g. “Bronchiectasis). For cases which were included in SCDM and had purely non-asthma and non-COPD diagnoses, we classified them in the “Neither” group. The reason for the expanded list of pre-defined diagnosis codes was to strengthen the case finding, which could then be filtered out during the subsequent analysis

^5^Refers to number of outpatient visits to respective institutions

## Discussion

We described the development of a near real-time integrated RWD database that includes demographic, clinical, laboratory and radiology data of 36,407 patients (as of December 31, 2019) with asthma and COPD across the spectrum from primary to tertiary care in our healthcare system. Data verification was performed and RWD database demonstrated near perfect agreement with the clinical EMR system. Having developed this data mart within an analytics platform simplifies the access to data via a drag-and-drop interface, rather than having to write SQL codes.

While several asthma and COPD databases already exist, the strength of the SCDM is that it links RWD from primary care to tertiary care and has a rich data capture for asthma and COPD that is near real-time. Data in the RWD are refreshed with a maximum of 24 h delay as the data refresh takes place overnight when the system utilization level is low. With an intentionally broad inclusion criteria and wide range of data elements, from demographics, clinical data, laboratory results to vaccinations and unscheduled visits, we are confident that it is sufficiently robust to meet most asthma and COPD research data needs. Table 6 shows a comparison of the SCDM (asthma only) with two other asthma databases, the International Severe Asthma Registry (ISAR) and Danish National Database for Asthma (DNDA) [4, 31, 32].

**Table 6:** Comparison of SCDM with International Severe Asthma Registry (ISAR) and Danish National Database for Asthma (DNDA)

	SingHealth COPD and Asthma Data Mart (SCDM)^1^	International Severe Asthma Registry (ISAR) [31]	Danish National Database for Asthma (DNDA) [32]
*Summary*			
Country	Singapore	International	Netherlands
No. of patients	29,730^2^	12,764^3^	> 300,000
Type of patients	Asthma and COPD, Primary care and specialist care, of all ages	Severe asthma with GINA Step 5, or Step 4 and uncontrolled asthma; 18 years and above only	Asthma patients age 6 and 44 years (excludes COPD)
Aim	To link primary care and tertiary care data To support health services research, facilitate conduct of pragmatic trials and studies To aid clinical decision making and inform policy changes in asthma and COPD in Singapore	To generate a centralized severe asthma dataset that would permit data to be shared seamlessly between countries and institutions, to ultimately gain better insight into severe asthma on a global scale	To collect the data on all patients treated for asthma in Denmark and to monitor asthma occurrence, the quality of diagnosis, and management
*Strengths and limitations*			
Strength	Automated data capture Weekly updates Broad range of data	Multi-national database Structured data fields with good data quality	Nation-wide database Automated data capture
Limitation	Currently limited to two healthcare institutions Some data captured in free-text requiring additional effort to clean before analysis	Manual method of data entry via an electronic data capture system Only patients with severe asthma	Only includes patients from 6 to 44 years old
*Availability of selected information*			
Medical history	Yes	Yes	Not stated^4^
Smoking history	Yes	Not stated^4^	Yes
Asthma control	Yes	Yes	Not stated^4^
Lung function test	Yes	Yes	Yes
Asthma medications	Yes	Yes	Yes
Reason for asthma medication switch (if any)	No (but can be mined from free-text medical history)	Yes	Not stated^4^
Non-asthma medication	Yes	Not stated^4^	Not stated^4^
Vaccination	Yes	Not stated^4^	Not stated^4^
Unscheduled visit	Yes	Not stated^4^	Not stated^4^

^1^Only comparing patients with asthma

^2^As of 31-Dec-2019

^3^https://isaregistries.org/ (Accessed: 15-Nov-2022)

^4^Not stated in respective database website or article

As our health system is based on geographical regions, it allows us to serve a captive population of patients who tend to seek care within the same health system. This provides researchers with the opportunity to use relatively more complete longitudinal data to study the disease and care trajectories of asthma and COPD patients as they move across the care chain, from primary care to specialist and acute care. A previous study on this health system showed that among the patients with stable chronic diseases, there were on average approximately 1.6 times more primary care visits as compared to specialist outpatient clinics visits [33]. The registry can further serve as a basis for determining computable phenotypes [34] such as frequent exacerbators, high risk (of poor outcome) patients, fixed obstruction and type 2 high inflammatory phenotype in an Asian population.

With the heavy investments in developing the ETL pipelines, we also designed the SCDM with flexibility and sustainability in mind. For this, we deliberately chose to perform minimal transformation to preserve the raw data and minimize information loss. Unlike specific disease or national registries that combine and transform raw data to derive composite variables, our database consists of almost completely raw data in their original format. The registry adopted the same classification as the raw data, and followed the International Classification of Diseases, ICD-9 and ICD-10 [35], and the Systematized Nomenclature of Medicine-Clinical Terms (SNOMED-CT) [36] coding standards. At the time of the study, Singapore adopted the Australian-refined Diagnosis Related Groups (AR-DRG) version 6 coding system [37]. Although not using a common data model (CDM), such as Sentinel, Observational Medical Outcomes Partnership (OMOP) and Patient Centred Outcomes Research Network (PCORNet), may make our data less linkable with data from other databases, we felt that the trade-off was in favour of generalizability of the data to meet a wide variety of definitions [38–41]. Amongst the various classification systems used, mappings exist between them to ensure the interpretability of results across multiple systems and globally across time. Furthermore, as the healthcare CDM space is still actively developing, we will have the option of migrating our database to a CDM [42].

Minimal filtering of the data was done as we attempted to capture the complete dataset that is available throughout the clinical processes. For example, we chose to import all medications prescribed for a patient, including non-asthma related medications, instead of filtering them based on a pre-selected list of asthma-related medications. This endowed the SCDM with the following advantages: (1) the flexibility to select medications of interest to their own study; (2) the capability to study effects and associations with non-asthma medications, and (3) the adaptability to include any new asthma and non-asthma related medications that may be prescribed in future without the need to update the underlying ETL pipelines.

Although agile methodologies are gaining in popularity in IT development space, we elected a hybrid methodology where the waterfall project plan is required to secure the resources for milestone delivery and to ensure governance requirements are duly complied. Some of the requirements to determine the cohort and data elements were well-defined and amenable to a waterfall methodology whilst within the design and development process, we have adopted the agile methodology for the refinement and implementation of the requirements [43, 44]. The uncertain requirements inherent in the design of a registry which leverages on clinical and operational data requires flexibility in efficient requirement changes [25]. The hybrid framework also allowed us to perform robust data verification that adheres to national and organizational data security policies at the final phases of the SCDM development process. Limited by organizational and data governance constraints, whilst requiring the need for flexibility through close stakeholders’ engagement to refine the data requirements, we have adopted a hybrid waterfall-agile approach towards the development of the SCDM [27].

Our RWD database is not without its limitations. Although it currently includes patients with asthma and COPD follow-up at SGH RCCM specialist clinics in the tertiary hospital, it does not include those who are only followed-up with other departments such as Internal Medicine, Occupational Medicine, or those who only visited the Accident and Emergency Department (A&E) within the same hospital, and were not referred to the SGH RCCM. Also, although the data mart contains rich clinical details, a significant proportion of this is in free-text format which requires additional data mining tasks before the data can be analysed. One example is the smoking status data where almost half was not available from structured data input fields. With the continual effort to encourage the adoption of standardized clinical templates for asthma and COPD, we hope to improve the quality of data capture. Furthermore, the standardization of semi-structured text formats will further enable us to make use of natural language processing (NLP) algorithms to derive relevant information from the textual data. It is envisioned that we could augment the registry with NLP capabilities to improve data completeness.

Moving ahead, as the next phase in the SDG-CARE collaboration, we will leverage the SCDM in several areas. One immediate area is to develop interactive dashboards that will be able to provide a real-time overview of the key statistics in SCDM, monitor routine practice and for clinical decision support. In terms of clinical research, the team has embarked on a project using SCDM data to develop a model that uses routinely available data in primary care to predict asthma exacerbations. This will support identification of at-risk patients such that earlier and more resource-intensive interventions may be applied for this group. By working with SCDM data which is already routinely captured in the EMR, the team will be able to more easily deploy the model for use. The team also intends for the SCDM to influence public health policies, and is using the real-world data to investigate the impact of guideline non-conformance, such as yearly influenza vaccinations, on clinical outcomes, such as visits to emergency or hospitalizations for pneumonia. Findings from this may potentially result in guideline changes or lend support to tighter compliance. Further down, we also envision that the SCDM will provide the foundation for RWD collection for impactful, large-scale pragmatic clinical trials, akin to the applications from the Salford Lung Study [45].

In parallel, we will also work towards iteratively enhancing the SCDM. In the next phase, we will look toward including data from the only public paediatric and maternity tertiary hospital in Singapore—KK Women’s and Children Hospital (KKWCH). This will open up the potential to observe long-term trajectory of asthma from paediatric to adulthood and to perform more in-depth studies on determinants of poor outcomes.

## Conclusion

We described the development of a RWD database for asthma and COPD in the largest public health care system in Singapore, spanning primary care to specialist and acute hospital care. By adopting a systematic process, we were able to ensure that it was robust, valid and applicable. This RWD database provides a unique opportunity for clinical and health services research in asthma and COPD, which can ultimately improve the care delivered to our patients.

## Supplementary Information

**Additional file 1: Supplementary Materials:** [1] List of data elements, and; [2] Procedure for external parties to obtain data from the SingHealth COPD and Asthma Data Mart (SCDM).

## Data Availability

Data from the SingHealth COPD and Asthma Data Mart (SCDM) may be made available on reasonable request. The process for external parties to obtain the data are outlined in Additional file 1: Annex A.

## Abbreviations

A&E: Accident & Emergency Department
ACOS: Asthma-COPD overlap syndrome
ACT: Asthma control test
BMI: Body mass index
BP: Blood pressure
CDM: Common data model
CAT: COPD assessment test
Clindoc: Sunrise Clinical Manager clinical document
COPD: Chronic obstructive pulmonary disease
CPRD: Clinical Practice Research Datalink
CT: Computerized tomography
CXR: Chest X-ray
DNDA: Danish National Database for Asthma
eHints: Electronic Health Intelligence System
EMR: Electronic medical records
ETL: Extract, transform and load
FEV1: Forced expiratory volume in the first second
FVC: Forced vital capacity
GINA: Global Initiative for Asthma
GOLD: Global Initiative for Chronic Obstructive Pulmonary Disease
GSK: GlaxoSmithKline
HES: Hospital Episode Statistics
ICD-10: 10Th revision of the International Classification of Diseases
ICU: Intensive care unit
IHiS: Integrated Health Information System
ISAR: International Severe Asthma Registry
IT: Information technology
KKWCH: KK Women’s and Children Hospital
mMRC: Modified Medical Research Council score
NLP: Natural language processing
OBIEE: Oracle Business Intelligence Enterprise Edition
OMOP: Observational Medical Outcomes Partnership
PCORNet: Patient Centred Outcomes Research Network
PCV13: Pneumococcal conjugate vaccine
PEFR: Peak expiratory flow rate
PPSV23: Pneumococcal polysaccharide vaccine
RCCM: Department of Respiratory and Critical Care Medicine
RWD: Real-world data
RWE: Real-world evidence
SCDM: SingHealth COPD and Asthma Data Mart
SCM: Sunrise Clinical Manager
SDG-CARE: SingHealth-Duke-GSK COPD and Asthma Real-World Evidence
SGH: Singapore General Hospital
SHP: SingHealth Polyclinics
SNOMED-CT: SNOMED Clinical Terms
UAT: User acceptance testing
URD: User requirements document
WAAP: Written asthma action plan
