# Supplementary material for: Development of a real-world database for asthma and COPD: The SingHealth-Duke-NUS-GSK COPD and Asthma Real-World Evidence (SDG-CARE) collaboration
Source: BMC Med Inform Decis Mak. 2023 Jan 9;23:4. doi: 10.1186/s12911-022-02071-6 (PMC9830781; doi:10.1186/s12911-022-02071-6)
Supplement: Supplementary file 1 — Additional file 1: Supplementary Materials: [1] List of data elements, and; [2] Procedure for external parties to obtain data from the SingHealth COPD and Asthma Data Mart (SCDM). [file 12911_2022_2071_MOESM1_ESM.docx]

**Supplementary Material** (Development of a Real-World Database for Asthma and COPD: The SingHealth-Duke-NUS-GSK COPD and Asthma Real-World Evidence (SDG-CARE) collaboration)

**Table 1.** Data elements in the SingHealth COPD and Asthma Data Mart (SCDM).

| **No.** | **Data element** | **Folder** |
| --- | --- | --- |
| 1 | Patient ID | Patient Demographics |
| 2 | Patient Name |  |
| 3 | Date of Birth |  |
| 4 | Gender |  |
| 5 | Race |  |
| 6 | Nationality |  |
| 7 | Marital Status |  |
| 8 | Postal Code |  |
| 9 | Death Date |  |
| 10 | Death Reason |  |
| 11 | Cohort Institution Code | Master List^1^ |
| 12 | Cohort Visit No |  |
| 13 | Cohort Visit Date |  |
| 14 | Cohort Diagnosis |  |
| 15 | Problem Institution Code | Problem List^2^ |
| 16 | Problem Case No |  |
| 17 | Problem Visit No |  |
| 18 | Problem Code^*^ |  |
| 19 | Problem Description |  |
| 20 | Year of Diagnosis |  |
| 21 | Problem Status |  |
| 22 | Problem Type |  |
| 23 | Lab Institution Code | Laboratory Results |
| 24 | Lab Case No |  |
| 25 | Lab Visit No |  |
| 26 | Lab Test |  |
| 27 | Lab Test Abnormality Code |  |
| 28 | Lab Test Entered Date |  |
| 29 | Lab Test Lower Limit |  |
| 30 | Lab Test Lower Limit / Lab Test Upper Limit |  |
| 31 | Lab Test Unit |  |
| 32 | Lab Test Upper Limit |  |
| 33 | Lab Test Value |  |
| 34 | Radiology Institution Code | Radiology Reports |
| 35 | Radiology Case No |  |
| 36 | Radiology Visit No |  |
| 37 | Radiology Test |  |
| 38 | Radiology Test Abnormality Code |  |
| 39 | Radiology Test Entered Date |  |
| 40 | Radiology Test Report |  |
| 41 | Radiology Test Report Part Number |  |
| 42 | Prescribed Institution Code | Medications Prescribed |
| 43 | Prescribed Case No |  |
| 44 | Prescribed Visit No |  |
| 45 | Dosage |  |
| 46 | Dosage Unit |  |
| 47 | Duration |  |
| 48 | Duration Unit |  |
| 49 | Form |  |
| 50 | Frequency |  |
| 51 | Medication Order Name |  |
| 52 | Prescribed Date |  |
| 53 | Prescribed Stop Date |  |
| 54 | Medication Order Date |  |
| 55 | SGH Dosage Frequency |  |
| 56 | Dispensed Institution Code | Medications Dispensed |
| 57 | Dispensed Case No |  |
| 58 | Dispensed Visit No |  |
| 59 | Dispensed Date |  |
| 60 | Medication Name |  |
| 61 | Medication Class |  |
| 62 | Dispensed Quantity |  |
| 63 | Dispensed Quantity Unit |  |
| 64 | Dispensed Duration |  |
| 65 | Prescribed Quantity |  |
| 66 | Dispensed Duration Unit |  |
| 67 | Visit Institution Code | Visit Details^3^ |
| 68 | Case No |  |
| 69 | Visit No |  |
| 70 | Visit Date Time |  |
| 71 | Visit Doctor |  |
| 72 | Visit Specialty Code |  |
| 73 | Visit Specialty Desc |  |
| 74 | Visit Sub Specialty Code |  |
| 75 | Visit Sub Specialty Desc |  |
| 76 | Visit Type Code |  |
| 77 | Visit Type Desc |  |
| 78 | Case Type Code |  |
| 79 | Case Type Desc |  |
| 80 | Visit Diagnosis |  |
| 81 | Visit Diagnosis Desc |  |
| 82 | Visit Location |  |
| 83 | Visit Status |  |
| 84 | Intensive care unit (ICU) stay |  |
| 85 | Length of stay |  |
| 86 | Diagnosis Institution Code | Diagnosis^4^ |
| 87 | Diagnosis Case No |  |
| 88 | Diagnosis Visit No |  |
| 89 | Admit/Visit Date |  |
| 90 | Admit Specialty |  |
| 91 | Case Type Code |  |
| 92 | Case Type Desc |  |
| 93 | Diagnosis Code^#^ |  |
| 94 | Diagnosis Desc |  |
| 95 | Diagnosis Type |  |
| 96 | Diagnosis Activity Group Desc |  |
| 97 | Rescue Visit No | Rescue Therapy^5^ |
| 98 | Rescue Therapy Code |  |
| 99 | Rescue Therapy Desc |  |
| 100 | Rescue Therapy Count |  |
| 101 | Rescue Therapy Date |  |
| 102 | Referral Institution Code | Referrals^6^ |
| 103 | Referral Visit No |  |
| 104 | Referral ID |  |
| 105 | Referral Diagnosis |  |
| 106 | Referral Hospital |  |
| 107 | Referral Date |  |
| 108 | Referral Department |  |
| 109 | Referral Department Others |  |
| 110 | Referral Reason |  |
| 111 | Document Name | Clinical Document Metadata |
| 112 | Patient History | Patient History^7^ |
| 113 | COPD Hospitalization |  |
| 114 | Objective Diagnosis | Objective Diagnosis |
| 115 | Asthma control test (ACT) Adult 1 | Asthma Control Test |
| 116 | ACT Adult 2 |  |
| 117 | ACT Adult 3 |  |
| 118 | ACT Adult 4 |  |
| 119 | ACT Adult 5 |  |
| 120 | ACT Child 1 |  |
| 121 | ACT Child 2 |  |
| 122 | ACT Child 3 |  |
| 123 | ACT Child 4 |  |
| 124 | ACT Child 5 |  |
| 125 | ACT Child 6 |  |
| 126 | ACT Child 7 |  |
| 127 | ACT Total |  |
| 128 | COPD assessment test (CAT) 1 | COPD Assessment Test |
| 129 | CAT 2 |  |
| 130 | CAT 3 |  |
| 131 | CAT 4 |  |
| 132 | CAT 5 |  |
| 133 | CAT 6 |  |
| 134 | CAT 7 |  |
| 135 | CAT 8 |  |
| 136 | CAT Total |  |
| 137 | mMRC | Modified Medical Research Council Dyspnea Scale |
| 138 | GOLD Score | GOLD Score |
| 139 | BMI | Physical Measurements |
| 140 | Diastolic BP |  |
| 141 | Height |  |
| 142 | Oxygen Saturation |  |
| 143 | Pain Score |  |
| 144 | Pulse Rate |  |
| 145 | Respiratory Rate |  |
| 146 | Systolic BP |  |
| 147 | Temperature |  |
| 148 | Weight |  |
| 149 | Physical Findings | Physical Examination^7^ |
| 150 | PEFR Calculated | Peak Expiratory Flow Rate |
| 151 | PEFR Personal Best |  |
| 152 | Spirometry Date | Spirometry Results |
| 153 | FEV1 Pre |  |
| 154 | FEV1 Pre % |  |
| 155 | FVC Pre |  |
| 156 | FVC Pre % |  |
| 157 | FEV1/FVC Ratio Pre |  |
| 158 | FEV1 Post |  |
| 159 | FEV1 Post % |  |
| 160 | FVC Post |  |
| 161 | FVC Post % |  |
| 162 | FEV1/FVC Ratio Post |  |
| 163 | Change in FEV |  |
| 164 | Change in FEV % |  |
| 165 | Change in FVC |  |
| 166 | Change in FVC % |  |
| 167 | Pre/Post indicator |  |
| 168 | Bronchodilator Response |  |
| 169 | Bronchodilator Change Mil |  |
| 170 | Bronchodilator Change % |  |
| 171 | Smoking Habit | Smoking Status |
| 172 | Smoking Habit Notes |  |
| 173 | Smoking Habit Pack Years |  |
| 174 | Smoking Habit Started Year |  |
| 175 | Smoking Habit Stopped Year |  |
| 176 | Smoking Sticks Per Day |  |
| 177 | Influenza Given | Vaccinations Details^8^ |
| 178 | Influenza Date |  |
| 179 | Influenza Outside Date |  |
| 180 | Influenza Location |  |
| 181 | Influenza Not Done |  |
| 182 | Influenza Last Asked |  |
| 183 | Pneumococcal PCV13 Given |  |
| 184 | Pneumococcal PCV13 Date |  |
| 185 | Pneumococcal PCV13 Outside Date |  |
| 186 | Pneumococcal PCV13 Location |  |
| 187 | Pneumococcal PCV13 Not Done |  |
| 188 | Pneumococcal PCV13 Last Asked |  |
| 189 | Pneumococcal PPSV23 |  |
| 190 | Pneumococcal PPSV23 Date/Time |  |
| 191 | Pneumococcal PPSV23 Outside Date |  |
| 192 | Pneumococcal PPSV23 Location |  |
| 193 | Pneumococcal PPSV23 Not Done |  |
| 194 | Pneumococcal PPSV23 Last Asked |  |
| 195 | WAAP | Written Asthma Action Plan |
| 196 | Management Plan | Management Plan^7^ |
| 197 | Health/Asthma Counselling | Asthma Counselling |
| 198 | Reason |  |
| 199 | Pulmonary Rehab | Pulmonary Rehabilitation |

The data extraction is done in a two-step process. Firstly, the Master List is used to extract the ID for patients of interest. Then with this list of IDs, the other subject areas are used to extract the rest of the data elements of interest.

1. Problem list conditions were based on SNOMED Clinical Terms (SNOMED-CT) coding.
2. Visits details includes details such as visit date, visit location, and visit provider.
3. Diagnosis conditions were based on 10^th^ revision of the International Classification of Diseases (ICD-10) coding.
4. Rescue therapy is a protocol-based bronchodilator intervention administered at the polyclinic for patients assessed to have asthma exacerbation.
5. Referrals made from SingHealth Polyclinics to tertiary hospitals, including Singapore General Hospital.
6. Patient history, physical examination findings and management plan were captured as free-text data.
7. For influenza and pneumococcal vaccinations only.

* SNOMED Clinical Terms (SNOMED-CT) codes.

# 10^th^ revision of the International Classification of Diseases (ICD-10) codes.

Abbreviations: COPD = Chronic obstructive pulmonary disease, GOLD = Global Initiative for Obstructive Lung Disease

**Annex A.** Outline of procedure for external parties to obtain data from the SingHealth COPD and Asthma Data Mart (SCDM).

The following sequence of steps outline the procedure for external parties to obtain data from the SingHealth COPD and Asthma Data Mart (SCDM). External parties refer to individuals or organizations that are not employed by or subsidiaries of SingHealth respectively. External parties interested to obtain data from the SCDM will need to collaborate with an investigator from SingHealth in order to gain access to the data. The procedure is aligned with organizational and national data protection policies. This procedure is correct as of this writing, but may be subject to change. (Non-external parties will follow organizational policies to obtain the data.)

Procedure for obtaining data for external parties

**Step 1**: External parties interested to obtain data from the SCDM should email SingHealth Health Services Research Centre (HSRC) – [hsr@singhealth.com.sg](mailto:hsr@singhealth.com.sg). The email should contain:

- Name of requestor and affiliated organization
- Reason for intent to collaborate
- List of data elements requested (anonymized/not anonymized)
- Preferred investigator from SingHealth (if any)

**Step 2**: SingHealth HSRC will direct the request to the registry owners and suitable investigators who may be interested to collaborate.

**Step 3**: Upon identifying an investigator from SingHealth, the external party and SingHealth investigator will enter into and sign a research collaboration agreement (RCA). This documents includes the objectives of the research project, obligations of each party and how the data will be shared.

**Step 4**: The SingHealth investigator will obtain all the necessary approvals (e.g. ethics board and data access).

**Step 5**: The SingHealth investigator will receive the data and share with external party in compliance to the RCA and approvals in Step 4.
